# Negative Venous Leg Ultrasound in Acute Pulmonary Embolism: Prevalence, Clinical Characteristics and Predictors

**DOI:** 10.3390/diagnostics12020520

**Published:** 2022-02-17

**Authors:** Mattes Becher, Thomas Heller, Sarah Schwarzenböck, Jens-Christian Kröger, Marc-André Weber, Felix G. Meinel

**Affiliations:** 1 Institute of Diagnostic and Interventional Radiology, Pediatric Radiology and Neuroradiology, University Medical Centre Rostock, 18057 Rostock, Germany; mattes.becher@uni-rostock.de (M.B.); thomas.heller@med.uni-rostock.de (T.H.); jens-christian.kroeger@med.uni-rostock.de (J.-C.K.); marc-andre.weber@med.uni-rostock.de (M.-A.W.); 2 Department of Nuclear Medicine, University Medical Centre Rostock, 18055 Rostock, Germany; sarah.schwarzenboeck@med.uni-rostock.de

**Keywords:** acute pulmonary embolism, deep venous thrombosis, duplex ultrasound, compression ultrasound

## Abstract

The purpose of this study was to investigate the prevalence, clinical characteristics, and predictors of negative venous leg ultrasound in acute pulmonary embolism (PE). We retrospectively analyzed a cohort of 168 patients with acute PE (median age 73 years, 44% women) evaluated with complete venous leg ultrasound. A multivariate logistic regression analysis was performed to identify the independent predictors of negative venous ultrasound in acute PE. Venous leg ultrasound was negative for deep venous thrombosis (DVT) in 78 patients (46.4%). Patients with negative venous ultrasound were less likely to have a history of DVT (7.7% vs. 20.0%, *p* = 0.0273) and had significantly lower D-dimer levels (median 2.5 vs. 6.2 mg/dL *p* < 0.0001). Negative venous ultrasound was more frequent in PE diagnosed with V/P-SPECT than in PE diagnosed with CT (66.2% vs. 34.0%, *p* < 0.0001). The prevalence of negative venous ultrasound increased with more peripherally located PE (29.5% for central/lobar, 43.1% for segmental, and 60.6% for subsegmental PE, *p* = 0.0049). For the multivariate analysis, a diagnosis of PE with V/P-SPECT rather than CT (OR 3.2, *p* = 0.0056) and lower D-dimer levels (OR 0.94, *p* = 0.0266) were independent predictors of negative venous ultrasound. In conclusion, venous leg ultrasound was negative for DVT in almost half of patients with acute PE. Negative venous ultrasound was more common in patients with no history of DVT, lower D-dimer levels, PE diagnosed with V/P-SPECT rather than CT, and more peripherally located PE.

## 1. Introduction

Acute pulmonary embolism (PE) is thought to originate from lower extremity deep venous thrombosis (DVT) in most cases. Predisposing factors for venous thromboembolism include cancer, previous DVT, inherited hypercoagulable conditions, and inflammatory diseases [1,2,3]. Patients with acute PE are typically referred for venous duplex ultrasound of the legs to diagnose DVT as the source of PE. However, it is surprisingly frequent in clinical routine for venous leg ultrasound to be negative in patients with newly diagnosed acute PE.

In studies dating back to the 1980s and 1990s, the prevalence of DVT in patients with newly diagnosed PE has been extremely variable, ranging from 13 to 93% [4]. These studies used the historical standard tests of pulmonary angiography and planar ventilation/perfusion (V/P)-scintigraphy to diagnose PE. The prevalence of DVT in these studies was substantially higher when venography was used to diagnose DVT (71–93%) than with compression ultrasound (13–29%) [4].

Due to the enormous technical advances since then, CT pulmonary angiography has replaced pulmonary angiography as the de facto clinical gold standard for the diagnosis of PE [1], and V/P imaging is now predominantly performed in the form of V/P-SPECT (single photon emission computed tomography) [5]. Similarly, comprehensive ultrasound (including compression and Doppler) has replaced diagnostic venography for the diagnosis of DVT [1,6].

Therefore, the purpose of this study was to investigate the prevalence, clinical characteristics, and predictors of negative venous leg ultrasound in acute PE with state-of-the-art technology. By systematically analyzing the predictors of negative venous leg ultrasound, we aim to shed light on the possible explanations for PE without DVT.

## 2. Materials and Methods

### 2.1. Ethical Approval, Study Design, and Patient Selection

**Figure 1:**
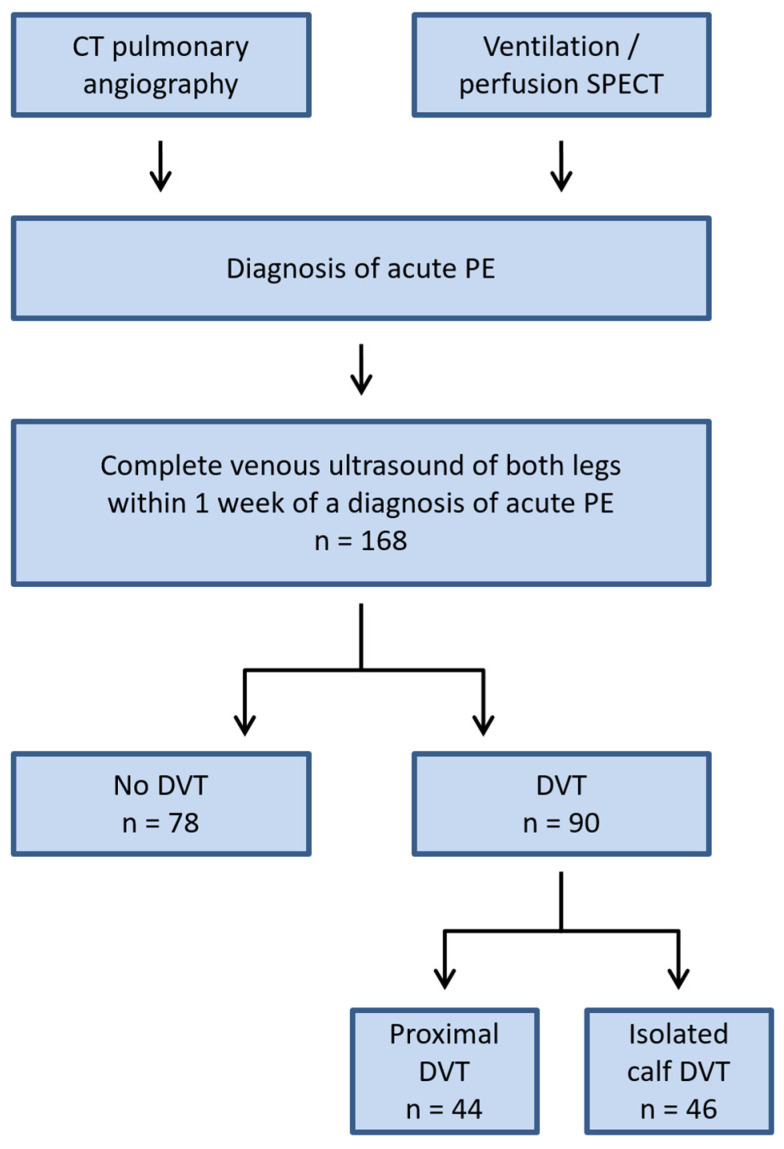
Flow chart of patient cohort.

This retrospective cohort study was performed with institutional review board approval (registration number A2022-030) and a waiver of informed consent. We analyzed all patients who were examined with venous ultrasound of the legs at our institution in 2014 for suspected DVT in the setting of a recently diagnosed acute PE (duplex ultrasound within 7 days of PE diagnosis; Figure 1). During 2014, two senior radiologists with extensive experience in vascular ultrasound (T.H. and J.-C.K.) almost exclusively performed all venous ultrasound examinations. Thus, this year was chosen for the purpose of our study. We identified eligible patients through a retrospective query of our radiology information system (Centrictiy 5.0, GE Healthcare, Chicago, IL, USA).

### 2.2. Ultrasound Technique

All venous ultrasound examinations were performed on a Toshiba Aplio XG SSA 770A system (Toshiba Medical Systems Corporation, Otawara, Japan). In the setting of acute PE, we always performed a complete venous ultrasound of both legs. Examinations were performed by board-certified and subspecialized radiologists. Our protocol for venous leg ultrasound included compression ultrasound, color Doppler, and spectral Doppler, and this has been previously described in detail [7].

### 2.3. Analysis of Radiology Reports and Clinical Data

Radiology reports of all evaluations were retrospectively reviewed by a medical student (M.B.) for the presence and location of DVT. Patients were classified as having proximal DVT if any portion of the DVT was in the iliac, femoral, and/or popliteal veins. Isolated calf DVT was defined as DVT limited to veins below the knee.

A review of the electronic patient charts was performed to record age, gender, presenting symptoms (leg pain, leg swelling, difference in leg circumference, and redness), risk factors (active malignancy; previous DVT; and known inherited hypercoagulable conditions such as Factor V Leiden, prothrombin gene mutations, deficiencies of antithrombin, protein C, or protein S), and D-Dimer levels.

### 2.4. CT Technique

CT pulmonary angiographies were performed on a 64-slice CT scanner (Aquilion 64, Toshiba Medical Systems Corporation, Otawara, Japan), as clinically indicated. Per institutional standard at the time of CT examinations, 70–80 mL of contrast media (Imeron 400 mg/mL, Bracco, Milan, Italy) were injected intravenously with a flow of 3–4 mL/s. Bolus-triggering in the main pulmonary artery was used to start the scan.

### 2.5. V/P-SPECT Technique

Ventilation and perfusion (V/P)-SPECT was performed according to the guidelines of the European Association for Nuclear Medicine (EANM) [5,8]. For the ventilation study, the patients inhaled about 30 MBq of ^99m^Tc-Technegas [9] for the perfusion study, and approximately 200 MBq of ^99m^Tc- MAA (MAA Sol, GE Healthcare) were injected intravenously immediately after completion of the ventilation study. The V/P images were acquired on a SPECT/CT (Symbia T6, Siemens Healthineers, Erlangen, Germany). Acquisitions were performed in step and shoot mode (60 projections per camera head, 3° angular resolution, scan time per projection 20 s (ventilation scan), and 10 s (perfusion scan)). SPECT series were reconstructed using a 3D-OSEM algorithm (8 iterations, 15 subsets, and Flash 3D) with compensation for depth-dependent collimator response followed by low-pass filtering. Perfusion SPECT was accompanied by a low dose CT (120 kVp, 50 mAS) used for morphological correlation and attenuation correction of the perfusion SPECT data. No scatter correction was applied.

### 2.6. Reporting of V/P SPECT/CT Studies

The V/P SPECT/CT studies were evaluated in accordance with the EANM guideline for ventilation/perfusion scintigraphy [5,8]. PE was diagnosed in case of at least one lobar or segmental vascular type mismatched defect (perfusion defect with preserved ventilation), or two sub-segmental vascular mismatches. PE was excluded if perfusion was normal and in the case of non-vascular type mismatches, matched defects, or reverse mismatches (preserved perfusion but absent ventilation). As recommended by the EANM guidelines, classification was not based on probability categories, but the findings were strictly categorized into “PE yes” and “PE no”, respectively. The localization of findings was given. V/P SPECT/CT studies were interpreted independently by two physicians (with at least one specialist in nuclear medicine) using reconstructed V- and P-images with standard SPECT/CT visualization software (Hermes Hybrid Viewer 2.2, Hermes Medical Solutions, Stockholm, Sweden).

### 2.7. Statistical Analysis

Commercially available software (GraphPad Prism, version 9.0.0, GraphPad Software Inc., La Jolla, CA, USA) was used for the statistical analysis. The median and interquartile range were calculated for the numerical parameters and compared between groups using the Mann−Whitney test. Frequencies and proportions were calculated for the categorical data. The distribution of categorical variables between groups was compared using Fisher’s exact test (for two groups) or Chi-square test (for three groups), as appropriate. Uni- and multivariate logistic regression analyses were performed to identify the independent predictors of the negative venous ultrasound. All predictors with a significant association in the univariate analysis were entered into the multivariate model. *p*-Values of <0.05 were considered to indicate statistical significance.

## 3. Results

### 3.1. Patient Characteristics

Our patient cohort included 168 patients with a recent (<7 days) diagnosis of acute PE referred for venous leg ultrasound to evaluate for DVT. The median age was 73 years. Seventy-four patients (44%) were women. Risk factors for venous thromboembolism included a history of DVT in 24 patients (14.3%) and active malignancy in 14 patients (8.3%).

### 3.2. Comparison of Patients with DVT vs. Patients without DVT on Ultrasound

The venous leg ultrasound was negative for DVT in 78 patients (46.4%; Table 1 and Figure 1). There were no differences in age or gender between patients with and without DVT upon ultrasound. Expectedly, leg symptoms including leg swelling (10.3% vs. 21.1% *p* = 0.0614), leg pain (12.8% vs. 15.6%, *p* = 0.6636), circumference difference (2.6% vs. 8.9%, *p* = 0.1079), and redness (1.3% vs. 4.4%, *p* = 0.3740) were less common in patients without DVT than in patients with DVT, but the differences were not statistically significant. Patients with a negative venous ultrasound were less likely to have a history of DVT (7.7% vs. 20.0%, *p* = 0.0273) and had significantly lower D-dimer levels (median 2.5 vs. 6.2 mg/dL, *p* < 0.0001).

The flow chart illustrates the study design and patient cohort included. CT = computed tomography; DVT = deep venous thrombosis; PE = pulmonary embolism; SPECT = single photon emission computed tomography.

### 3.3. Location of DVT on Duplex Ultrasound

Among the 90 patients with DVT, 44 patients (48.9%) had proximal DVT and 46 patients (51.1%) had isolated calf DVT. Proximal DVTs involved the inferior vena cava in 1 patient, the iliac veins in 5 cases, the common femoral vein in 15 patients, the deep femoral vein in 5 cases, the superficial femoral vein in 31 cases, and the popliteal vein in 30 cases (29 patients had proximal DVT in more than one of the above proximal veins). Isolated lower leg DVTs were seen in the posterior tibial veins in 22 patients, fibular veins in 36 patients, and muscle veins (gastrocnemius or soleus) in 12 patients (21 patients had isolated lower leg DVT in more than one of the above calf veins).

### 3.4. Comparison of Patients with CT-Diagnosed and V/P-Diagnosed PE

The diagnosis of acute PE had been established with CT in 103 patients (61.3%) and with V/P-SPECT in 65 patients (38.7%; Table 2). Patients diagnosed with CT were younger (median age 70 vs. 75 years, *p* = 0.0019) and had higher median D-dimer levels (5.0 vs. 3.2 mg/L, *p* = 0.0383) than the patients diagnosed with V/P-SPECT. The other characteristics were similar. Negative venous ultrasound was significantly more frequent in PE diagnosed with V/P-SPECT than in PE diagnosed with CT (66.2% vs. 34.0%, *p* < 0.0001). Both proximal DVT (15.4% vs. 33%, *p* = 0.0120) and isolated calf DVT (18.4 vs. 33%, *p* = 0.0505) were less frequently seen in PE diagnosed with V/P-SPECT than in PE diagnosed with CT.

### 3.5. Results of Duplex Ultrasound by Most Proximal Localization of PE

The most proximal location of PE on CT or V/P-SPECT was in the main or lobar pulmonary arteries in 44 patients (26.2%), in the segmental pulmonary arteries in 58 patients (34.5%), and in subsegmental pulmonary arteries in 66 patients (39.3%; Table 3). The prevalence of negative venous ultrasound increased with more peripherally located PE (29.5% for central/lobar PE, 43.1% for segmental PE, and 60.6% for subsegmental PE; *p* = 0.0049). In particular, the prevalence of proximal DVT decreased with more peripherally located PE (43.2% for central/lobar PE, 27.6% for segmental PE, and 13.6% for subsegmental PE; *p* = 0.0025). The prevalence of isolated calf DVT did not differ between these subgroups.

### 3.6. Predictors of a Negative Ultrasound Result

For the univariate analysis (Table 4), having no history of DVT (Odds Ratio 3.0, *p* = 0.0280), lower D-dimer levels (OR 0.93, *p* = 0.0066), PE diagnosed with V/P-SPECT rather than CT (OR 3.8, *p* < 0.0001), and more distal location of PE (OR 1.9, *p* = 0.0014) were significant predictors of a negative ultrasound result. For the multivariate analysis (Table 5), a diagnosis of PE with V/P-SPECT rather than CT (OR 3.2, *p* = 0.0056) and lower D-dimer levels (OR 0.94, *p* = 0.0266) were independent predictors of a negative venous ultrasound.

## 4. Discussion

Newly diagnosed PE without DVT on venous leg ultrasound is a common but poorly understood clinical scenario. Possible explanations for PE without DVT include complete embolization of lower extremity DVT, venous thromboembolism from uncommon sites (hepatic, renal, ovarian [10], neck, or upper extremity veins [11,12]), false-positive diagnosis of PE, false-negative venous leg ultrasound, in situ thrombosis in the pulmonary arteries [13], and complete resolution of lower extremity DVT due to anticoagulation therapy in the short time interval between the diagnosis of PE and venous leg ultrasound.

There is scarce contemporary literature on the frequency of PE without DVT. In an observational study of 574 patients with PE, no DVT was found in 148 patients (26%) [14]. A more recent analysis of 428 patients with PE found that complete bilateral compression ultrasound was negative in 29% of patients [15]. These previous studies reporting a lower prevalence of negative Duplex ultrasound included only patients with symptomatic PE. In our cohort, we included all patients with newly diagnosed PE including cases of PE incidentally detected on CT. This may have led to more peripheral PEs being diagnosed—and these are more frequently associated with a negative ultrasound. Sane and colleagues analyzed 63 cases of acute PE and found an absence of DVT in 50% of cases [3]. Thus, the 46% rate of negative ultrasound in our cohort is consistent with previous literature.

Velmahos and colleagues observed that among 46 trauma patients with PE, 85% did not have DVT of the pelvic, femoral, or popliteal veins [2]. In a similar cohort of 31 trauma patients who developed PE, van Gent and colleagues found that 39% did not have DVT on duplex sonography [13]. They hypothesized that PE in trauma patients may originate in situ as a local response to endothelial injury or inflammation, and may thus represent a distinct entity from venous thromboembolism.

It could be argued that the high prevalence of PE without DVT simply means that venous leg ultrasound often fails to locate the source of PE. While ultrasound has very high sensitivity and specificity for femoral and popliteal veins [6], more experience is required and the accuracy can be lower for calf veins [6,16] and pelvic veins [17]. In addition, DVT in uncommon sites (hepatic, renal, ovarian, neck, or upper extremity veins) will be missed by venous leg ultrasound. This hypothesis has been addressed by a unique study using whole-body MRI to search for DVT in patients with newly diagnosed PE [18]. Interestingly, even on whole-body MRI, no DVT was found in 56% of patients with PE [18]. This suggests that PE without DVT is a true and common clinical phenomenon rather than the result of a false-negative venous ultrasound.

The presence or absence of DVT in acute PE has important prognostic implications. In one study, 3-month mortality was 12.9% for PE with DVT and 4.6% for PE without DVT [14]. In a meta-analysis on this topic, the 30-day all-cause mortality was 6.2% in PE with DVT and 3.8% in PE without DVT (odds ratio 1.9) [19].

In our study, we systematically analyzed predictors of a negative venous ultrasound in patients with acute PE. Negative venous ultrasound was more common in patients with no history of DVT, lower D-dimer levels, PE diagnosed with V/P-SPECT rather than CT, and more peripherally located PE. In the multivariate analysis, a diagnosis of PE with V/P-SPECT rather than CT and lower D-dimer levels were independent predictors of a negative venous ultrasound. These findings are consistent with and go beyond previously published cohorts. Not surprisingly, leg symptoms of DVT are associated with DVT in acute PE [15]. In most studies, the peripheral location of PE was associated with the absence of DVT [3,13,15]. This was also observed in our cohort. The results are more mixed with regards to other predictors of a negative venous ultrasound. Female gender was associated with a negative ultrasound in some studies [14,15], but not in others [3,20]. Our finding that negative venous ultrasound in PE is more common in patients without a personal history of venous thromboembolism is supported by at least one study [14], although other authors did not find this association [3,15]. One study found that malignancy was more common in patients with PE without DVT than in patients with PE and DVT [20].

A novel finding of our study is that the rate of negative venous ultrasound in newly diagnosed PE was substantially higher for PE diagnosed with V/P-SPECT than in PE diagnosed with CT. In part, this may be explained by referral bias. Because CT is faster and more readily available in the emergency setting, patients with a more acute presentation and more severe symptoms are more likely to be investigated with CT, whereas patients in a stable condition may be more likely to undergo V/P scanning. Therefore, patients with larger and more central pulmonary emboli are more likely to be diagnosed with CT—and we know from our and previous studies [3,13,15] that a more central location of PE is associated with the presence of DVT. However, even on multivariate analysis adjusted for location of PE, PE diagnosed with V/P-SPECT rather than CT was a strong independent predictor of negative venous ultrasound in our cohort. One might hypothesize that ventilation/perfusion mismatch on V/Q-SPECT might be less specific for acute PE than the direct visualization of intravascular filling defects with CT. However, the specificity of V/P-SPECT is generally thought to be very high (96–98%) [5], thus it is unlikely that limited specificity of V/P-SPECT can explain this finding. There were differences in age and D-dimer levels between patients diagnosed with CT than patients diagnosed with V/P-SPECT. However, these differences do not plausibly explain the higher negative ultrasound rate in patients diagnosed with V/P-SPECT. In addition, PE diagnosed with V/P-SPECT rather than CT remained a strong independent predictor of negative venous ultrasound even when adjusted for D-dimer levels in the multivariate model. Ultimately, our data do not provide an explanation for why negative venous ultrasound is more common in PE diagnosed with V/P-SPECT rather than CT. Certainly, this observation warrants further research.

We acknowledge several limitations of our study. This was a single-center cohort study in a limited number of patients. Due to the retrospective nature of our dataset, we were not able to analyze patients with negative venous leg ultrasound for possible alternative sources of pulmonary embolism. There was no external reference standard to confirm the findings at the venous ultrasound. Thus, we cannot exclude that some cases of DVT were missed by venous ultrasound. However, this risk is mitigated in our study as complete venous ultrasound of both legs was performed by experienced radiologists in all patients. Furthermore, it would have been interesting to analyze the ultrasound findings for different risk strata of pulmonary embolism. Unfortunately, due to the retrospective nature of this study, we did not have reliable data on hemodynamic parameters, oxygen saturation, and right heart dysfunction for all patients, and therefore we could not perform reliable risk-stratification.

## 5. Conclusions

Venous leg ultrasound was negative for DVT in almost half of patients with acute PE. Negative venous ultrasound was more common in patients with no history of DVT, lower D-dimer levels, PE diagnosed with V/P-SPECT rather than CT, and more peripherally located PE.

## Figures and Tables

**Table 1:** Characteristics of the study population.

		All Patients (*n* = 168)		No DVT (*n* = 78)		DVT (*n* = 90)		*p*-Value
		N	%	N	%	N	%	
**Demographics**	Females	74	44%	37	47.4%	37	41.1%	0.4388
	Age in years, median (interquartile range)	73 (59–80)		75 (62–81)		73 (58–79)		0.3583
	BMI in kg/m^2^, median (interquartile range)	27.15 (24.5–32.2) [*n* = 132]		26.7 (23.6–32.1) [*n* = 61]		27.2 (25.2–32.3) [*n* = 71]		0.2250
**Presentation**	Leg pain	24	14.3%	10	12.8%	14	15.6%	0.6636
	Leg swelling	27	16.1%	8	10.3%	19	21.1%	0.0614
	Circumference difference	10	6%	2	2.6%	8	8.9%	0.1079
	Redness	5	3%	1	1.3%	4	4.4%	0.3740
	Any (of the above) leg symptoms	45	26.8%	17	18.9%	28	35.9%	0.2216
**Risk factors**	Inherited hypercoagulable conditions	1	0.6%	0	0%	1	1.1%	0.9999
	Active cancer	14	8.3%	6	7.7%	8	8.9%	0.9999
	Previous DVT	24	14.3%	6	7.7%	18	20%	**0.0273**
**Lab**	D-dimers in mg/L, median (interquartile range)	4.2 (2.0–9.7) [*n* = 138]		2.5 (1.3–4.5) [*n* = 58]		6.2 (3.4–12) [*n* = 80]		**<0.0001**
	Cardiac Troponin T in ng/mL, median (interquartile range)	0.026 (0.011–0.060) [*n* = 129]		0.017 (0.010–0.035) [*n* = 57]		0.030 (0.014–0.089) [*n*= 72 ]		0.0629

BMI = body mass index; DVT = deep venous thrombosis; *p*-values < 0.05 appear bold.

**Table 2:** Patient characteristics and results of duplex ultrasound by modality of PE diagnosis.

		All Patients (*n* = 168)		CT-Diagnosed Acute PE (*n* = 103)		V/P-Diagnosed Acute PE (*n* = 65)		*p*-Value
		N	%	N	%	N	%	
**Demographics**	Females	74	44%	43	41.7%	31	47.7%	0.5238
	Age in years, median (interquartile range)	73 (59–80)		70 (56–78)		75 (69–82)		**0.0019**
	BMI in kg/m^2^, median (interquartile range)	27.2 (24.5–32.2) [*n* = 132]		27.7 (24.8–32.8) [*n* = 85]		26.6 (24.5–31.3) [*n* = 47]		0.3166
**Risk factors**	Inherited hypercoagulable conditions	1	0.6%	1	1.0%	0	0%	1.0000
	Active cancer	14	8.3%	10	9.7%	4	6.2%	0.5695
	Previous DVT	24	14.3%	18	17.5%	6	9.2%	0.1760
**Lab**	D-dimers in mg/L, median (interquartile range)	4.2 (2.0–9.7) [*n* = 138]		5.0 (2.2–11) [*n* = 81]		3.2 (1.6–6.3) [*n* = 57]		**0.0383**
	Cardiac Troponin T in ng/ml, median (interquartile range)	0.026 (0.011–0.060) [*n* = 129]		0.029 (0.011–0.091) [*n* = 76]		0.025 (0.011–0.047) [*n* = 53]		0.6255
**Result of** **Ultrasound**	No DVT	78	46.4%	35	34%	43	66.2%	**<0.0001**
	DVT	90	53.6%	68	66%	22	33.8%	
	Proximal DVT	44	26.2%	34	33%	10	15.4%	**0.0120**
	Isolated Calf DVT	46	27.4%	34	33%	12	18.4%	**0.0505**

BMI = body mass index; CT = computed tomography; DVT = deep venous thrombosis; PE = pulmonary embolism; V/P = ventilation/perfusion imaging; *p*-values < 0.05 appear bold.

**Table 3:** Results of duplex ultrasound by most proximal localization of PE.

	All Patients (*n* = 168)		Central/ Lobar PE (*n* = 44)		Segmental PE (*n* = 58)		Subsegmental PE (*n* = 66)		*p*-Value
	N	%	N	%	N	%	N	%	
**No DVT**	78	46.4%	13	29.5%	25	43.1%	40	60.6%	**0.0049**
**DVT**	90	53.6%	31	70.5%	33	56.9%	26	39.4%	
**Proximal DVT**	44	26.2%	19	43.2%	16	27.6%	9	13.6%	**0.0025**
**Isolated calf DVT**	46	27.4%	12	27.3%	17	29.3%	17	25.8%	0.9065

DVT = deep venous thrombosis; PE = pulmonary embolism; *p*-values < 0.05 appear bold.

**Table 4:** Predictors of ultrasound negative for DVT (univariate analysis).

Predictor	Odds Ratio	95% Confidence Interval	*p*-Value
Age in years	1.003	0.983–1.024	0.3572
Female gender	1.293	0.702–2.389	0.4105
Absence of Any Leg symptoms	1.620	0.812–3.306	0.1757
No History of DVT	3.000	1.182–8.669	**0.0280**
D-Dimers (per 1 mg/L increment)	0.926	0.871–0.974	**0.0066**
PE diagnosed with V/P rather than CT	3.797	1.991–7.420	**<0.0001**
More distal location of PE on CT or V/P	1.927	1.298–2.913	**0.0014**

CT = computed tomography; DVT = deep venous thrombosis; PE = pulmonary embolism; V/P = ventilation/perfusion imaging; *p*-values < 0.05 appear bold.

**Table 5:** Predictors of ultrasound negative for DVT (multivariate analysis).

Predictor	Odds Ratio	95% Confidence Interval	*p*-Value
No History of DVT	1.787	0.614–5.744	0.3019
D-Dimers (per 1 mg/L increment)	0.941	0.887–0.989	**0.0266**
PE diagnosed with V/P rather than CT	3.242	1.421–7.580	**0.0056**
More distal location of PE on CT or V/P	1.479	0.848–2.598	0.1676

CT = computed tomography; DVT = deep venous thrombosis; PE = pulmonary embolism; V/P = ventilation/perfusion imaging; *p*-values < 0.05 appear bold.

## Data Availability

All supporting data are available from the corresponding author upon reasonable request.

## References

[1] Khan F., Tritschler T., Kahn S.R., Rodger M.A. (2021). Venous thromboembolism. Lancet.

[2] Velmahos G.C., Spaniolas K., Tabbara M., Abujudeh H.H., de Moya M., Gervasini A., Alam H.B. (2009). Pulmonary embolism and deep venous thrombosis in trauma: Are they related?. Arch. Surg..

[3] Sane M.A., Laukkanen J.A., Granér M.A., Piirilä P.L., Harjola V.-P., Mustonen P.E. (2019). Pulmonary embolism location is associated with the co-existence of the deep venous thrombosis. Blood Coagul. Fibrinolysis.

[4] Girard P., Musset D., Parent F., Maitre S., Phlippoteau C., Simonneau G. (1999). High prevalence of detectable deep venous thrombosis in patients with acute pulmonary embolism. Chest.

[5] Bajc M., Schümichen C., Grüning T., Lindqvist A., Le Roux P.-Y., Alatri A., Bauer R.W., Dilic M., Neilly B., Verberne H.J. (2019). EANM guideline for ventilation/perfusion single-photon emission computed tomography (SPECT) for diagnosis of pulmonary embolism and beyond. Eur. J. Nucl. Med. Mol. Imaging.

[6] Zierler B.K. (2004). Ultrasonography and diagnosis of venous thromboembolism. Circulation.

[7] Beller E., Becher M., Meinel F.G., Kröger J.-C., Rajagopal R., Höft R., Weber M.-A., Heller T. (2020). Prevalence and predictors of alternative diagnoses on whole-leg ultrasound negative for acute deep venous thrombosis. BMC Med. Imaging.

[8] Bajc M., Neilly J.B., Miniati M., Schuemichen C., Meignan M., Jonson B. (2009). EANM guidelines for ventilation/perfusion scintigraphy: Part 1. Pulmonary imaging with ventilation/perfusion single photon emission tomography. Eur. J. Nucl. Med. Mol. Imaging.

[9] Burch W.M., Sullivan P.J., McLaren C.J. (1986). Technegas--a new ventilation agent for lung scanning. Nucl. Med. Commun..

[10] Verde F., Johnson P.T. (2012). One not to miss: Ovarian vein thrombosis causing pulmonary embolism with literature review. J. Radiol. Case Rep..

[11] Levy M.M., Albuquerque F., Pfeifer J.D. (2012). Low incidence of pulmonary embolism associated with upper-extremity deep venous thrombosis. Ann. Vasc. Surg..

[12] Levy M.M., Bach C., Fisher-Snowden R., Pfeifer J.D. (2011). Upper extremity deep venous thrombosis: Reassessing the risk for subsequent pulmonary embolism. Ann. Vasc. Surg..

[13] van Gent J.-M., Zander A.L., Olson E.J., Shackford S.R., Dunne C.E., Sise C.B., Badiee J., Schechter M.S., Sise M.J. (2014). Pulmonary embolism without deep venous thrombosis: De novo or missed deep venous thrombosis?. J. Trauma Acute Care Surg..

[14] Sevestre M.-A., Quashié C., Genty C., Rolland C., Quéré I., Bosson J.-L. (2010). Clinical presentation and mortality in pulmonary embolism: The Optimev study. J. Mal. Vasc..

[15] Hirmerova J., Seidlerova J., Chudacek Z. (2018). The Prevalence of Concomitant Deep Vein Thrombosis, Symptomatic or Asymptomatic, Proximal or Distal, in Patients With Symptomatic Pulmonary Embolism. Clin. Appl. Thromb. Hemost..

[16] Kearon C., Julian J.A., Newman T.E., Ginsberg J.S. (1998). Noninvasive diagnosis of deep venous thrombosis. McMaster Diagnostic Imaging Practice Guidelines Initiative. Ann. Intern. Med..

[17] Stern J.-B., Abehsera M., Grenet D., Friard S., Couderc L.-J., Scherrer A., Stern M. (2002). Detection of pelvic vein thrombosis by magnetic resonance angiography in patients with acute pulmonary embolism and normal lower limb compression ultrasonography. Chest.

[18] van Langevelde K., Srámek A., Vincken P.W.J., van Rooden J.-K., Rosendaal F.R., Cannegieter S.C. (2013). Finding the origin of pulmonary emboli with a total-body magnetic resonance direct thrombus imaging technique. Haematologica.

[19] Becattini C., Cohen A.T., Agnelli G., Howard L., Castejón B., Trujillo-Santos J., Monreal M., Perrier A., Yusen R.D., Jiménez D. (2016). Risk Stratification of Patients With Acute Symptomatic Pulmonary Embolism Based on Presence or Absence of Lower Extremity DVT: Systematic Review and Meta-analysis. Chest.

[20] Schwartz T., Hingorani A., Ascher E., Marks N., Shiferson A., Jung D., Jimenez R., Jacob T. (2012). Pulmonary embolism without deep venous thrombosis. Ann. Vasc. Surg..

